# Clinical features and genetic analysis of a Brazilian patient with sitosterolemia: a case report

**DOI:** 10.20945/2359-4292-2024-0326

**Published:** 2025-05-31

**Authors:** Felipe Augusto Azevedo Leão, Leticia Ferreira Gontijo Silveira, Rodrigo Rezende Arantes, Milena Maria Moreira Guimarães

**Affiliations:** 1 Serviço de Endocrinologia, Departamento de Clínica Médica, Faculdade de Medicina, Universidade Federal de Minas Gerais, Belo Horizonte, MG, Brasil; 2 Serviço de Genética Médica, Faculdade de Medicina, Universidade Federal de Minas Gerais, Belo Horizonte, MG, Brasil

**Keywords:** Hypercholesterolemia, Lipid metabolism disorders, Genetics

## Abstract

Sitosterolemia is a rare genetic lipid disorder caused by mutations in the *ABCG5/ABCG8*, genes. It is characterized by plasmatic plant sterols accumulation, formation of tendon and tuberous xanthomas and early onset coronary artery disease. The differential diagnosis with other congenital dyslipidemias presents significant challenges. We describe a case of a male patient who presented with hypercholesterolemia and tendinous xantomas from the age of 5. The patient was born to consanguineous parents, with no family history of hypercholesterolemia. With the initial hypothesis of cerebrotendinous xanthomatosis, he was treated with chenodeoxycholic acid, which yielded no improvement. Over time, he developed persistent thrombocytopenia and arthralgia, and experienced an acute myocardial infarction at the age of 27. Genetic analysis revealed the previously known p.Trp361*mutation in homozygosity in the *ABCG8* gene and was negative for *CYP27A1* variants, associated with cerebrotendinous xanthomatosis. The subsequent introduction of a diet with vegetable fats restriction and administration of ezetimibe resulted in an excellent response. The diagnosis of congenital hypercholesterolemia is challenging due to the low prevalence and heterogenous presentation of the condition. This case underscores the importance of clinical suspicion and the confirmation of the molecular diagnosis for a precise therapeutic management.

## INTRODUCTION

Sitosterolemia is a rare congenital lipid metabolism disorder characterized by increased intestinal absorption and decreased biliary excretion of plant sterols and cholesterol, resulting in accumulation of very high serum concentrations of plant sterols, such as sitosterol, campesterol and stigmasterol (^1^,^2^). It is an autosomal recessive condition, caused by inactivating mutations in the genes encoding adenosine triphosphate-binding cassette transporters G5(*ABCG5*) and G8 (*ABCG8*) (^3^,^4^).

Typically, sitosterolemia presents with tendinous and tuberous xanthomas from an early age, with progressive development, and premature coronary atherosclerosis, mirroring the clinical presentation of familial hypercholesterolemia (^2^,^4^-^8^). However, the clinical manifestations may be highly heterogeneous and other clinical features such as hemolytic anemia, thrombocytopenia, splenomegaly, and arthralgia/arthritis may be present as well (^2^,^5^,^8^). While plasma β-sitosterol concentrations are elevated in all patients with sitosterolemia, the availability of this test is not widespread. The differential diagnosis with other congenital dyslipidemias is quite challenging, and it may be difficult to achieve a correct diagnosis based solely on the clinical and laboratorial features. The primary differential diagnoses include familial hypercholesterolemia and cerebrotendinous xanthomatosis (CTX) (^2^,^9^). The management of the patients involves dietary restriction of plant sterols and the use of lipid lowering drugs such as ezetimibe and bile acid sequestrant resins (^2^,^9^,^10^). We report here a case of sitosterolemia, in which the correct diagnosis and treatment were only possible after the molecular diagnosis.

## CASE REPORT

**Figure 1:**
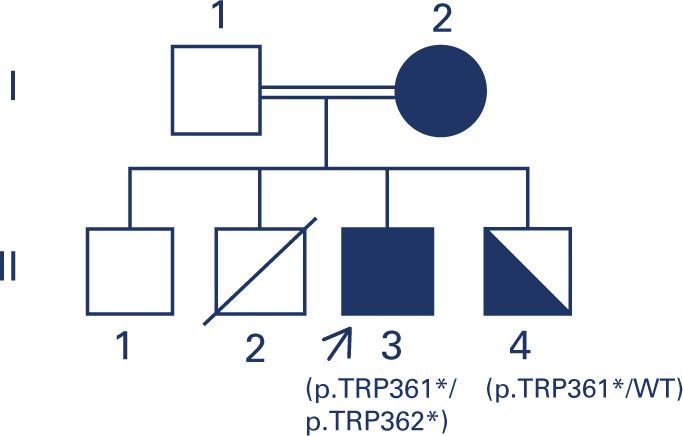
The pedigree of the proband (arrow) and his family. Members II-2 and II-3 were submitted to DNA analysis. The proband (II-2) was homozygous for the *ABCG8* mutation, and his brother (II-2), with mild dyslipidemia, was heterozygous for the same mutation. Solid symbol: affected family member; half-solid symbol: heterozygous carrier.

**Figure 2:**
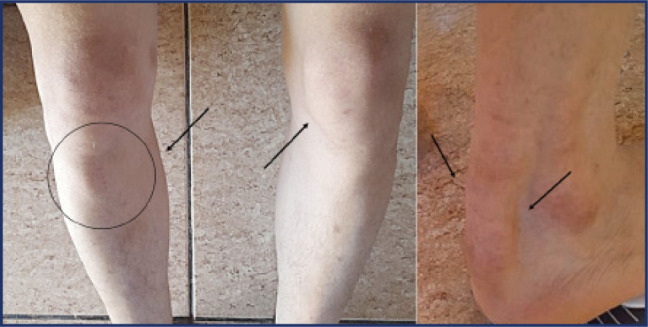
Tendinous xanthomas in the Achilles tendon and knee, respectively. Arrows highlight the lesions in tendons.

This study was approved by the ethics committee of *Universidade Federal de Minas Gerais* (UFMG) (# 6.127.386). Written informed consent was obtained from the patient for publication of his medical case details. A 17-year-old boy was referred to the Endocrinology Service in 2005 for evaluation of possible congenital dyslipidemia. He had a history of painful tendinous xanthomas since he was 5 years old, initially in the Achilles tendons, progressing to elbows, wrists, and knees, as well as polyarthralgia and hypercholesterolemia, especially due to elevated low-density lipoprotein (LDL)-cholesterol elevation; he had no other symptoms and otherwise unremarkable past medical history. The patient was born to consanguineous parents, and had three siblings, one healthy (**Figure 1**), one who died immediately after birth and another one diagnosed with Crohn’s disease and ankylosing spondylitis. No familial history of dyslipidemia or premature atherosclerosis was reported. At physical examination, he presented with yellowish plaques on the eyelids bilaterally, scaly lesions on elbows and knees and thickening of the Achilles and patellar tendons (**Figure 2**), with no other abnormalities. Laboratory tests are listed in **Table 1**. The measurement of plasmatic phytosterols was not available. Magnetic resonance imaging (MRI) of the brain was performed due to diagnostic suspicion for CTX and revealed discrete symmetric changes in the signal intensity of the cerebellar dentate nuclei, periventricular frontoparietal white matter, corticospinal tracts, and globus pallidus. Knees and Achilles tendon MRI showed signals of chronic tendinopathy suggestive of xanthomas, confirmed by Achilles tendon biopsy.

**Table 1:** Laboratory data of the patient during the follow-up

Parameter (unit)	Before treatment					After treatment			
	2005	2009	2011	2015	2017	2018	2019	2022	2023
Hemoglobin (g/100mL)		15.6		16	15		14.4	14.6	15,1
Total leukocytes (× 109/L)		5,600		4,600	7,000		4,600	6,060	4,290
Platelets (×109/L)		78	46	150	57		65	132	116
Total cholesterol (mg/dL)	243	223	153	223	169	173	179	160	132
HDL-cholesterol (mg/dL)	50	30	59	100	60	58	48	48	58
LDL-cholesterol (mg/dL)	177	172	81	108	91	98	113	94	56
Triglycerides (mg/dL)	83		65	73	89	86	90	91	90
AST (U/L)		20		73	34	30	32	25	
ALT (U/L)		23		35	34	25	27	30	
GGT (U/L)		29		241	30	69			
ALP (U/L)		318		7	85	30	40		
Creatinine (mg/dL)			1.12			0.93			

HDL: high density cholesterol; LDL: low density cholesterol; AST: aspartate transaminase; ALT: alanine transaminase; GGT: gamma-glutamyl transferase; ALP: alkaline phosphatase.

With the initial presumptive diagnosis of CTX, treatment with chenodeoxycholic acid was initiated but interrupted due to lack of response, diarrhea, epigastric pain, pruritus, and hyperbilirubinemia. Throughout the irregular medical follow-up, he developed persistent thrombocytopenia, with no defined etiology at the time. In 2015, at the age of 27, he developed an acute myocardial infarction, requiring coronary stent implantation in the anterior descending artery, and secondary prevention therapy was initiated with rosuvastatin 10 mg daily, acetylsalicylic acid 100 mg daily, and clopidogrel 75 mg daily.

**Figure 3:**
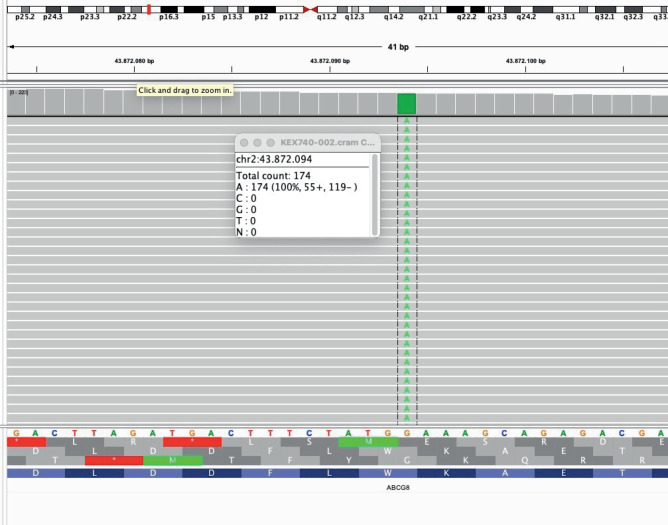
Genetic sequencing tracing confirming the homozygous p.Trp361* mutation in the *ABCG8* gene.

**Figure 4:**
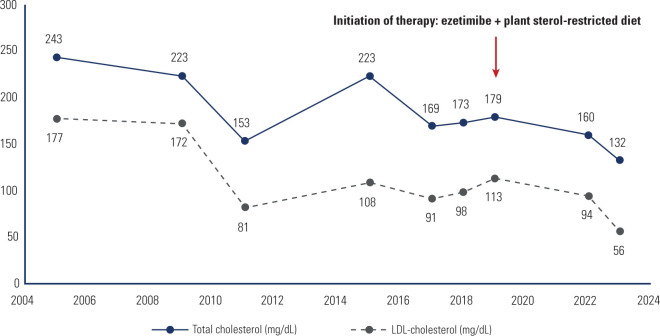
Evolution of total cholesterol and low-density lipoprotein-cholesterol levels during follow-up. LDL: low density cholesterol.

After a multidisciplinary discussion in 2018, genomic DNA was extracted in order to sequence *CYP27A1* and *ABCG8* genes, associated with CTX and sitosterolemia, respectively. No mutations in *CYP27A1* were detected. A homozygous nonsense mutation was identified in the *ABCG8* gene, p.Trp361*, which was already known to be associated with sitosterolemia (**Figure 3**). His unaffected brother was heterozygous for the mutation. Following the molecular diagnosis, he has been managed with a plant sterol-restricted diet and the selective intestinal cholesterol absorption inhibitor ezetimibe, showing good clinical and metabolic response and an increment in platelet count (**Table 1 and Figure 4**). A coronary angiotomography was performed in 2021, revealing the absence of new plaques or coronary stenosis and a patent stent implanted in the middle third of the anterior descending artery.

## DISCUSSION

Plant sterols, including sitosterol, campesterol, and stigmasterol are molecules naturally present at low levels in plant-based foods such as fruits, vegetables, nuts and cereals. They are structurally very similar akin to cholesterol but differ by the presence of an ethyl group in sitosterol and stigmasterol or a methyl group in campesterol at C-24 of the sterol side chain (^11^). While approximately 50% of dietary cholesterol is absorbed, less than 5% of plant sterols are absorbed in individuals in physiological conditions. In normal conditions, the dietary intake of plant sterols competitively inhibits cholesterol absorption, thereby helping to reduce plasmatic cholesterol levels (^2^,^5^,^11^). *ABCG5/ABCG8* proteins form heterodimers that act as sterol transport complexes, which play a key role in the regulation of whole-body sterol trafficking, by eliminating sterols via the biliary tree as well as the intestinal tract (^12^). Normally, *ABCG5/ABCG8* transporters are responsible to shuttle absorbed plant sterols back into the intestinal lumen, allowing for the preferential incorporation of cholesterol esters into the chylomicrons (^11^). In sitosterolemia, dysfunctional forms of the *ABCG5/ABCG8* transporters lead to the accumulation of plant sterols due to increased absorption coupled with reduced hepatic excretion, subsequently increasing plasma cholesterol levels, which contributes to increased atherogenesis and complications such as coronary and carotid artery disease (^1^,^2^,^13^).

In 2000, Berge and cols. (^3^) identified *ABCG5* and *ABCG8* loss-of-function mutations in nine patients, thus elucidating the molecular pathogenesis of sitosterololemia. Since then, roughly 80 variants have been reported in about 120 patients across the medical literature, with an almost uniform distribution between *ABCG5* and* ABCG8*. Oligogenic cases involving combined mutations in both *ABCG5* and *ABCG8* or in conjunction with other familial hypercholesterolemia genes have also been reported (^2^).

Sitosterolemia is an extremely rare condition with an estimated prevalence of 1 in 200,000 individuals (^2^,^14^). However, this figure is likely an underestimation due to the lack of available laboratory and genetic diagnostic methods (^7^,^14^,^15^). *ABCG5* mutations appear to be more prevalent among patients of Asian descent, while *ABCG8* mutations in gene are seemingly more common in other populations (^7^). Brinton and cols. evaluated 207.926 patients with hypercholesterolemia (LDL-cholesterol > 190 mg/dL), finding that approximately 0.3% exhibited plasma β-sitosterol concentrations consistent with sitosterolemia (^16^). In another study conducted in China from 2002 to 2018, researchers reported that 26 cases were diagnosed in Hong Kong, Taiwan, and China, with 73% of these cases involving *ABCG5* mutations (^17^). Nevertheless, the prevalence of sitosterolemia and the genetic background are unknown in the Brazilian population.

Despite its global rarity, understanding the epidemiology of sitosterolemia is crucial due to the significant individual harm in cases that are not promptly diagnosed and treated. The main clinical manifestations of sitosterolemia include xanthomas at various sites, hypercholesterolemia, premature coronary heart disease, and, less frequently, thrombocytopenia, splenomegaly, and arthritis/arthralgia (^5^). Although the patient discussed herein exhibited all these manifestations throughout the disease, a correct diagnosis was only achieved following genetic analysis. The early diagnosis of sitosterolemia is imperative for the immediate initiation of appropriate treatment to prevent life-threatening complications (^16^). Delays in diagnosis due to the unavailability of plasma β-sitosterol and genetic testing undeniably contributed to the adverse progression of the patient’s condition, ultimately leading to an acute myocardial infarction. Notably, the patient’s clinical features were initially investigated separately, with a cohesive diagnosis only later established.

Following the molecular diagnosis, the therapeutic strategy was directed towards plant sterols and cholesterol restriction, alongside with ezetimibe administration (**Table 1, Figure 4**). Ezetimibe is established as one of the standard therapies, in view of sitosterolemia pathophysiology, characterized by increased absorption of plant sterols in the intestine and decreased secretion in the liver. Ezetimibe acts as an inhibitor of both cholesterol and phytosterols cholesterol intestinal absorption. After its rapid absorption, ezetimibe undergoes glucuronidation in the intestine and liver, and is mobilized to the brush border of the enterocytes, where alongside with its metabolites, it blocks the absorption of dietary and biliary sources of cholesterol, through the reduction of specific cholesterol-transporter enzymes in the gut (^17^). Despite the known unresponsiveness of individuals with sitosterolemia to statins, treatment with rosuvastatin was continued in this patient due to a previous coronary event.

In conclusion, we described a case of sitosterolemia with a homozygous mutation in the *ABCG8* gene. The case reported here underscores the challenges in clinically diagnosing sitosterolemia and differentiating it from other forms of early-onset or familial hypercholesterolemia, emphasizing the importance of molecular diagnosis for targeted therapeutic management. The diagnosis of sitosterolemia is complicated by its low prevalence, heterogeneous presentation and the limited availability of essential laboratory tests. Hence, the prompt initiation of the correct therapeutic approach is crucial to preventing the development of symptoms and the use of inadequate therapies, which can severely impair the quality of life and, more critically, increase the risk of life-threatening cardiovascular complications.
